# Special Issue “Lentiviral Vectors”

**DOI:** 10.3390/v14071492

**Published:** 2022-07-08

**Authors:** Yasuhiro Takeuchi

**Affiliations:** 1 Division of Infection and Immunity, University College London, London WC1E 6BT, UK; y.takeuchi@ucl.ac.uk; 2 Biotherapeutics and Advanced Therapies, Scientific Research and Innovation, Medicines and Healthcare Products Regulatory Agency, South Mimms EN6 3QG, UK

Lentiviral vectors (LV) have been developed upon knowledge accumulated in the virology field, in particular intensive research on HIV biology since its discovery in 1983. They are a driving force for the development of advanced therapies in medicine and are routinely used in basic biology research, and to support responses to public health threats. The Special Issue of *Viruses* titled “Lentiviral Vectors” has published eleven articles responding to the call “for reviews and original papers in this wide area of research related to lentiviral vectors” announced in January 2020. I am delighted that these articles have been offered by researchers from various fields, from molecular biology, virology, immunology and clinical science to engineering. They cover various aspects related to LV development and application.

LV systems using viral elements (vector genome backbone and particle structural proteins) originated from lentiviral species other than the most commonly used HIV-1 were reviewed in [1]. Another type of modification in these lentiviral elements is the engineering to turn the vector into a non-integrating or integration-deficient LV (NILV/IDLV), which was reviewed in [2]. Two research papers using IDLV describe their utility in cancer immunotherapy [3] and gene-mediated anti-viral antibodies [4]. Control of the expression level of transgenes is necessary to achieve optimal expression levels in the right cells and tissues. Various strategies to tackle this were discussed in [5]. Another part of vector particles, the envelope, is a focal point for alteration and modification as in the so-called pseudotyping practice. Pseudotyping of LV with various non-lentiviral envelopes for gene transfer purposes [6] and serological studies and testing [7] was reviewed. Such versatility of LV is exemplified in the research paper testing chimeric envelope proteins between two different viruses on gene transfer to neuronal cells [8]. Another review on pseudotyping using various envelope proteins focused on gene transfer into hematopoietic cells, an important gene therapy target cell type [9]. Among these cells, T cells are the LV gene transfer target in commercialized CAR-T cell therapies, and the current practice and prospect of manufacturing CAR-T cells were reviewed in [10]. Additionally, with commercial application in mind, LV bioprocessing was extensively reviewed in [11].

This Special Issue should attract a wide range of readers including those who are not regular *Viruses* readers. I have a small regret that there is no article on actual clinical applications of LV. Regardless, this variety of topics including LV and CAR-T manufacturing will be of interest to clinical researchers and therapy developers as well as others. I hope this Special Issue will enhance cross-field interaction and collaboration and help advance LV systems and all scientific fields benefitting from LV technology.
